# Editorial: Unraveling molecular mechanisms of neurodegenerative disease development: insights from stem cell-derived 2D and 3D *in vitro* models

**DOI:** 10.3389/fmolb.2026.1802206

**Published:** 2026-03-04

**Authors:** Stefania Scalise, Matteo Becatti, Valeria Lucchino

**Affiliations:** 1 Department of Experimental and Clinical Medicine, Magna Graecia University of Catanzaro, Catanzaro, Italy; 2 Department of Experimental and Clinical Biomedical Sciences “Mario Serio”, University of Firenze, Firenze, Italy

**Keywords:** 3D *in vitro* models, neurodegenerative disease, neurodegenerative disease development, stem cell, stem cell-based 3D systems

Neurodegenerative diseases continue to place an enormous burden on patients, caregivers, and healthcare systems, while effective strategies to prevent or slow disease progression remain limited. Within this context, the Research Topic “*Unraveling Molecular Mechanisms of Neurodegenerative Disease Development: Insights from Stem Cell-Derived 2D and 3D in vitro Models*” brings together seven contributions (four original research articles and three review articles) that illustrate how advances in molecular biology, experimental modeling, and translational thinking are reshaping our understanding of these disorders. By encompassing well-defined cellular and *in vivo* experimental systems alongside integrative and conceptual analyses, these studies pursue a common objective of dissecting the molecular pathways underlying neuronal dysfunction and degeneration while advancing experimental strategies and modeling tools that improve mechanistic insight and support the rational development of future therapeutic approaches.

In an original research study, Kirss et al. provide compelling experimental evidence that α-lipoic acid can normalize copper homeostasis in a neuronal cell model of Alzheimer’s disease by slowly increasing intracellular copper without inducing toxicity in contrast to fast-acting synthetic copper ionophores “*α-Lipoic Acid: A Potential Regulator of Copper Metabolism in Alzheimer’s Disease*”, thereby highlighting the importance of kinetic control in metal-based therapeutic strategies and reinforcing the relevance of neuronal cell models for dissecting metalloneurochemistry. Complementing this experimental work, Seneff and Kyriakopoulos propose a broad unifying theory in which deuterium overload, mitochondrial dysfunction, cardiolipin oxidation, and copper-histidine interactions collectively contribute to amyloidogenesis and neurodegeneration, reframing protein aggregation as a potentially protective metabolic response “*Deuterium Trafficking, Mitochondrial Dysfunction, Copper Homeostasis, and Neurodegenerative Disease*” and emphasizing mitochondria-centered mechanisms that are highly relevant to stem cell-derived neuronal and organoid models.

At the interface between molecular targeting and therapeutic discovery, Hassan et al. use structure-guided virtual screening and molecular dynamics simulations to identify phytochemical inhibitors of cathepsin B with high binding stability and favorable ADMET profiles “*Structure-guided Virtual Screening Reveals Phytoconstituents as Potent Cathepsin B Inhibitors*”, providing rationally designed candidate molecules and demonstrating how computational approaches can inform downstream testing in cellular and 3D disease models. Experimental neuroprotection is further explored by Taha et al., who show that silymarin-loaded nanoliposomes markedly attenuate monosodium glutamate-induced cerebellar toxicity *in vivo* by activating the PI3K/AKT pathway, reducing oxidative stress and inflammation, and preserving Purkinje cell integrity “*Neuroprotective Effect of Silymarin-loaded Nanoliposomes Against Monosodium Glutamate-Induced Cerebellar Motor Deficit*”, underlining the importance of delivery systems in maximizing the efficacy of neuroprotective molecules. A critical perspective is provided by Lee et al., who systematically review antioxidant interventions in facial nerve injury and show that while oxidative stress modulation consistently affects molecular and histological endpoints, functional recovery remains highly dependent on injury model, timing, and dose “*The Role of Antioxidants in Facial Nerve Injury*”, thereby offering important methodological lessons that extend to neurodegenerative disease modeling more broadly.

Expanding the scope of molecular investigation beyond classical brain-derived samples, Cavenaghi et al. introduce a non-invasive saliva-based transcriptomic and metagenomic framework to study neuroimmune and mitochondrial responses to music exposure in autism spectrum disorder “*Decoding the Peripheral Transcriptomic and Meta-genomic Response to Music in Autism Spectrum Disorder via Saliva-based RNA Sequencing*”, revealing coordinated changes in gene expression, co-expression networks, and microbial taxa and illustrating how peripheral omics can inform brain-related mechanisms and therapeutic monitoring.

Finally, Tan et al. review recent advances in three-dimensional bioprinting of the blood-brain barrier, detailing how bioinks, spatial architecture, and multicellular integration can be optimized to generate physiologically relevant human BBB models “*Advances in 3D Bioprinting for Modeling the Blood-Brain Barrier in Neurodegenerative Diseases*”, a critical component for modeling neurodegeneration, drug permeability, and neurovascular dysfunction in stem cell-based 2D and 3D systems.

Taken together, these studies exemplify the multidimensional approach promoted by this Research Topic, demonstrating that meaningful progress in understanding neurodegenerative disease mechanisms will arise from integrating molecular detail, innovative *in vitro* platforms, computational and omics technologies, and carefully contextualized therapeutic strategies.

